# Task Difficulty Modulates the Disrupting Effects of Oral Respiration on Visual Search Performance

**DOI:** 10.5334/joc.77

**Published:** 2019-08-02

**Authors:** Naoto Yoshimura, Fumiya Yonemitsu, Fernando Marmolejo-Ramos, Atsunori Ariga, Yuki Yamada

**Affiliations:** 1 Graduate School of Human-Environment Studies, Kyushu University, JP; 2 Japan Society for the Promotion of Science, Tokyo, JP; 3 Center for Change and Complexity in Learning, University of South Australia, AU; 4 Graduate School of Integrated Arts and Sciences, Hiroshima University, JP; 5 Faculty of Arts and Science, Kyushu University, JP

**Keywords:** Attention, Visual search, Action

## Abstract

Previous research has suggested that oral respiration may disturb cognitive function and health. The present study investigated whether oral respiration negatively affects visual attentional processing during a visual search task. Participants performed a visual search task in the following three breathing conditions: wearing a nasal plug, wearing surgical tape over their mouths, or no modification (oral vs. nasal vs. control). The participants searched for a target stimulus within different set sizes of distractors in three search conditions (orientation vs colour vs conjunction). Experiment 1 did not show any effect due to respiration. Experiment 2 rigorously manipulated the search efficiency and found that participants required more time to find a poorly discriminable target during oral breathing compared with other breathing styles, which was due to the heightened intercept under this condition. Because the intercept is an index of pre-search sensory processing or motor response in visual search, such cognitive processing was likely disrupted by oral respiration. These results suggest that oral respiration and attentional processing during inefficient visual search share a common cognitive resource.

## Introduction

Humans breathe consciously or unconsciously, inhaling oxygen through the mouth or nose. Neither breathing style is problematic if the purpose of breathing is only to inhale oxygen. Nevertheless, humans typically breathe through the nose. Many studies have reported that oral respiration styles, such as sleep-disordered breathing, have various long-term negative effects on human health (Pevernagie, De Meyer, & Claeys, 2005; Saint Martin, Sforza, Roche, Barthélémy, & Thomas-Anterion, 2015; Young, Finn, & Kim, 1997). Other studies have suggested that chronic oral respiration negatively impacts the academic performance of children and facial development (Jefferson, 2010; Kuroishi, Garcia, Valera, Anselmo-Lima, & Fukuda, 2015).

Moreover, recent studies have reported that breathing rate and phase manipulation influences cognitive function. For example, slow breathing reduced pain intensity and negative emotions (Arch & Craske, 2006; Zautra, Fasman, Davis, & Craig, 2010). Moreover, breathing phases modulate fear discrimination and memory retrieval (Nakamura, Fukunaga, & Oku, 2018; Zelano et al., 2016). Furthermore, brain activation studies have investigated whether oral and nasal respiration influence brain function. A study using near-infrared spectroscopy (NIRS) suggested that oral respiration increased the oxygen load in the prefrontal cortex (Sano, Sano, Oka, Yoshino, & Kato, 2013). Given this finding, it is plausible to assume that short-term breathing manipulation affects cognitive function.

Although various studies have examined the long-term effects of breathing on human performance, few studies have examined its short-term effects. Individuals with rhinitis must engage in cognitive activities, such as visual attentional tasks during driving, while their choice of breathing style is temporarily restricted. Therefore, it is important to investigate what happens in this situation. Visual attention is an essential function controlling the selection of objects from the field of view. Apparently, this function is indispensable for all cognitive activities of daily life. Considering the negative effects of mouth breathing (Pevernagie et al., 2005; Saint Martin et al., 2015; Young et al., 1997), we can predict that attentional function is disrupted by mouth breathing. Therefore, it is very important to investigate how breathing styles impact attentional function during our daily activities, which has relevance to workplace safety. Thus, the present study used visual search tasks to examine how short-term breathing affects visual attention.

Visual search tasks are effective for investigating visual attention. During visual search tasks, participants search among distractors for a target defined by specific feature dimensions. When observers are engaged in visual search tasks, the allocation of attention, which can be modulated by various factors (e.g., search history; Maljkovic & Nakayama, 1994), is responsible for their performance. In general, visual attention is controlled in a goal-directed, stimulus-driven, or goal-directed and stimulus-driven manner (Miller & Buschman, 2013). Stimulus-driven attention is directed, in a bottom-up manner, in descending order of saliency (Theeuwes, 1992, 2010), whereas goal-directed attention is top-down controlled by an observer’s goal or strategy (Bacon & Egeth, 1994). To put it simply, in the context of the target-distractor relationship, the dissimilarity of the distractors and target is likely to encourage stimulus-driven attentional allocation, whereas similarity is likely to encourage goal-directed attentional allocation (Vecera, Cosman, Vatterott, & Roper, 2014); however, there may be other modulating factors (Awh, Belopolsky, & Theeuwes, 2012; Theeuwes, 2018; Wolfe & Horowitz, 2017). Neural activity has been measured to examine the difference between stimulus- and goal-driven attention. A previous study suggested that the prefrontal cortex is engaged during inefficient visual searching associated with top-down attention control (Anderson et al., 2007). In fact, previous studies have suggested that the prefrontal cortex is directly involved in top-down attentional control (Bichot, Heard, DeGennaro, & Desimone, 2015; Buschman & Miller, 2007; Shimamura, 2000). In addition, attentional capture paradigm studies have suggested that the frontal cortex is involved in the top-down control of visual attention during search tasks (de Fockert, Rees, Frith, & Lavie, 2004; de Fockert & Theeuwes, 2012).

Indeed, many studies have suggested various factors responsible for the allocation of visual attention. However, converging evidence indicates that prefrontal cortex activity underlies goal-directed allocation of attention, which can be reflected in an inefficient search. An inefficient visual search is a phenomenon in which search performance slows as the number of distractors increases in response to sequential goal-directed allocation of attention to target items. Prefrontal cortex activities also underlie breathing styles, especially oral respiration (Sano et al., 2013). Given that overlapping brain regions are activated by not only oral respiration, but also top-down attentional control, it is plausible to assume that mouth breathing and top-down attentional control could interact. This issue is the focus of this study. We predicted that oral respiration affects the control of visual attention, particularly when observers are engaged in inefficient visual search tasks.

In visual search tasks, the slope and intercept are used as indices of attentional processing during visual search. The slope of the reaction time (RT) × set size function reflects the search efficiency, or serial deployment of attention from item to item (Wolfe & Horowitz, 2017). The intercept reflects sensory processing, decision making, and motor response. To address our hypothesis that oral respiration affects visual search performance, we examined whether oral respiration alone modulates search slope and intercept values compared with nasal respiration alone and natural respiration (i.e., nasal and oral respiration).

## Experiment 1

### Methods

#### Participants

Fifteen graduate and undergraduate students at Kyushu University (eight women; mean age: 20.73 years, *SD* = 2.61, age range: 19–27) participated in the experiment. All participants provided written informed consent in accordance with the Declaration of Helsinki. They received a 1500-yen cash voucher as compensation. The ethics committee of Kyushu University approved the study protocol (approval number: 2017-004).

#### Apparatus and Stimuli

**Figure 1:**
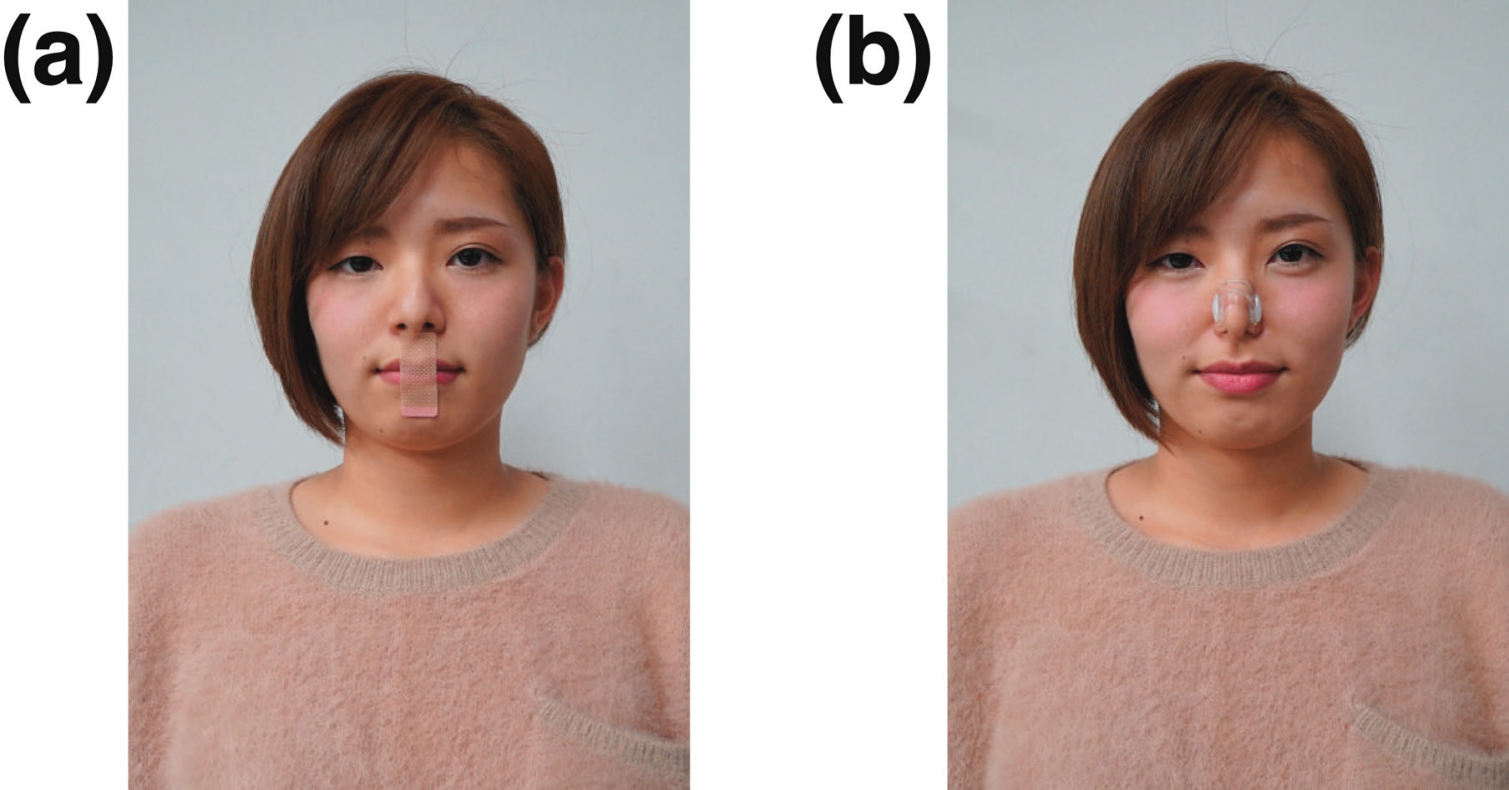
Experimental manipulation. A participant wearing a **(a)** strip of surgical tape or **(b)** nasal plug.

Participant breathing styles were manipulated using a 21 mm × 52 mm piece of surgical tape (SY32; Nichiban, Tokyo, Japan) and nasal plug (85ZN75001; Mizuno, Osaka, Japan), as shown in Figure 1. The experiment was conducted on multiple PCs using the open source library “jsPsych” for stimulus presentation and data collection (de Leeuw, 2015).

During the visual search task, either nine or 31 stimuli were presented at random positions in an imaginary grid. The grid pattern consisted of 36 imaginary squares (six rows × six columns). Each imaginary square was 101 × 101 pixels.

#### Procedure

**Figure 2:**
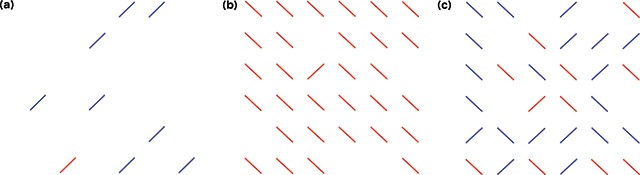
Three sample displays from Experiment 1. **(a)** Different colour search for target, and distractor in set size 9. **(b)** Different orientation search for target, and distractor in set size 31. **(c)** Different orientation and colour (conjunction) search for target, and distractor in set size 31.

The experiment was conducted in a quiet room. The participants initially conducted 18 visual search trials for practice. Subsequently, they performed the main visual search task under the following three conditions that manipulated their breathing style: 1) a condition in which nose breathing was encouraged by applying tape to the mouth (nasal respiration), 2) a condition in which mouth breathing was encouraged by wearing a nasal plug on the nose (oral respiration), and 3) natural breathing (control) without wearing equipment (see Figure 1). The experimenters ensured that participants did not breathe differently from the established breathing styles. The experimenters also checked that the nose plug did not block visibility during oral respiration. Each participant’s viewing distance was adjusted to 50 cm from the screen, and participants were instructed to perform the task while maintaining this viewing distance using a chin rest. Participants were instructed to search for a target among homogeneous or heterogeneous distractors. They were instructed to press the “F” key if the target was present and “J” key if the target was absent, or vice versa, which was counterbalanced across participants. When the participants pressed either the F or J key, their RT was measured. In the visual search task, the target was always a red bar tilted 45° to the right. The task consisted of three conditions, including a colour condition in which the target and distractors had different colours, an orientation condition in which the target and distractors had different orientations, and a conjunction condition that combined both features. In the colour condition, blue bars tilted 45° to the right were presented as distractors (homogeneous distractors). In the orientation condition, red bars tilted 45° to the left were presented as distractors (homogenous distractors). Red bars tilted 45° to the left and blue bars tilted 45° to either the left or right were distractors in the conjunction condition (heterogeneous distractors; see Figure 2). Thus, in the former two conditions, the participants were engaged in feature search tasks, whereas, in the last condition, they were engaged in conjunction (or serial) search tasks that require goal-directed attentional control. In addition, in half the trials, the target stimulus was absent. Thus, the experiment consisted of the following 12 conditions: two set sizes (9 and 31), three target-defining features (colour, orientation, and conjunction), and target presence (present and absent) conditions. All independent variables were manipulated within subjects using a repeated-measures design. Each condition was repeated 10 times. Thus, the total trial number was 120. The trials were divided into three blocks of 40 trials based on the search type, and breaks were given after every 20 trials. The order of the three different breathing style blocks was randomized for each participant. Participants could not predict the set size and target presence of the next trial. The 120-trial experimental set was repeated three times to measure the breathing styles (nasal respiration, oral respiration, and control) in a randomized order, yielding 360 trials.

#### Design and Statistical Analyses

Data were only analysed for correct responses in each target-presence condition and were log-transformed (RT_log10_). RT_log10_ outliers were defined as values that were 1.5 interquartile differences above the third quartile or below the first quartile of the respective empirical distribution. Outliers in the data were determined for each participant × breathing style × target-defining feature × set size cell and were excluded from correct RT_log10_ trials. The data were analysed using a repeated-measure analysis of variance (ANOVA) of the RT with factors, including breathing style (nasal, oral, or control breathing), target-defining feature (colour, orientation, or conjunction) and set size (9 or 31), to assess the effects of breathing style on visual attention. To analyse slopes and intercepts, we fitted linear regression equations to the mean log-transformed RTs plotted against the set size in the experimental conditions for each participant. The slopes and intercepts were also analysed by a repeated-measure ANOVA of the RT_log10_s of the breathing style and target-defining feature variables. We list the means of the slope (ms/item) and intercept (ms) from Experiment 1 in Table 1.

**Table 1:** Mean Search Slopes (ms/item) and Intercepts (ms) with Standard Errors (in Brackets) in Experiment 1.

Slope						

	Present			Absent		

Breathing style	Colour	Orientation	Conjunction	Colour	Orientation	Conjunction

Control	1.08 (0.54)	0.39 (0.43)	9.82 (1.99)	0.34 (0.49)	2.24 (0.94)	26.46 (2.88)
Nasal	0.42 (0.36)	–0.53 (0.37)	9.08 (1.38)	0.22 (0.69)	2.43 (0.60)	27.31 (4.40)
Mouth	0.62 (0.42)	0.16 (0.49)	9.40 (2.41)	–0.21 (0.51)	4.38 (1.09)	24.27(3.73)
**Intercept**						

Control	463.68 (13.93)	532.91 (21.47)	626.10 (37.74)	472.39 (21.53)	554.87 (26.23)	422.41 (36.97)
Nasal	488.00 (23.45)	546.03 (17.96)	669.21 (36.75)	513.07 (34.21)	561.98 (18.16)	418.3 (37.31)
Mouth	483.22 (23.44)	550.45 (17.41)	696.56 (55.22)	513.15 (32.00)	572.53 (18.16)	462.06 (50.82)

### Results

**Figure 3:**
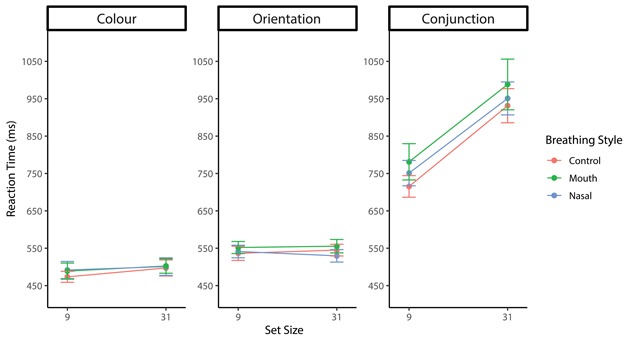
Mean RTs for each target-feature plotted as a function of breathing style and set size in target-present trials for Experiment 1. Error bars represent the standard error of the mean.

**Figure 4:**
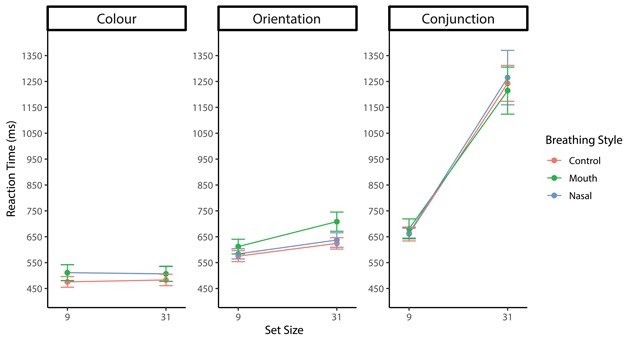
Mean RTs for each target-feature plotted as a function of breathing style and set size in target-absent trials for Experiment 1. Error bars represent the standard error of the mean.

The actual RTs are plotted in Figure 3 and 4 as an overview of the pattern of RTs. Repeated-measures ANOVA for target-present trials revealed significant main effects of the target-defining feature (*F*(2, 28) = 252.61, *p* < .001, η_p_^2^ = .95) and set size (*F*(1, 14) = 63.66, *p* < .001, η_p_^2^ = .82). In addition, there was a significant interaction between the target-defining feature and set size (*F*(2, 28) = 75.45, *p* < .001, η_p_^2^ = .84). However, main effects of the breathing style (*F*(2, 28) = 1.79, *p* = .19, η_p_^2^ = .11) and interactions between the breathing style and target-defining feature (*F*(4, 56) = 1.00, *p* = .41, η_p_^2^ = .01) or breathing style and set size (*F*(4, 56) = 0.43, *p* = .65, η_p_^2^ = .03) were not significant.

Repeated-measures ANOVA for target-absent trials revealed significant main effects of the target-defining feature (*F*(2, 28) = 128.44, *p* < .001, η_p_^2^ = .90) and set size (*F*(1, 14) = 137.10, *p* < .001, η_p_^2^ = .91). There was a significant interaction between the target-defining feature and set size (*F*(2, 28) = 86.61, *p* < .001, η_p_^2^ = .86). However, there were no significant main effects of the breathing style (*F*(2, 28) = 1.79, *p* = .19, η_p_^2^ = .11) and interaction between the breathing style and set size (*F*(4, 56) = 0.43, *p* = .65, η_p_^2^ = .03). Although there was a significant interaction between the breathing style and target-defining feature (*F*(4, 56) = 2.60, *p* < .05, η_p_^2^ = .16), multiple comparisons showed no significant difference between the respiratory conditions (*t*s < 0.24, *p*s > .04). Since the overall error rate was very low (2.72%), a speed-accuracy trade-off was not observed.

#### Slopes

The search slopes of the target-present trials were analysed using repeated-measures ANOVA with breathing style × target-defining feature as factors. This analysis showed a significant main effect of the target-defining feature (*F*(2, 28) = 75.45, *p* < .001, η_p_^2^ = .84), but the main effects of the breathing style (*F*(2, 28) = 0.43, *p* = .65, η_p_^2^ = .03) and interaction (*F*(4, 56) = 0.08, *p* = .99, η_p_^2^ = .01) were not significant. Post hoc comparisons demonstrated that the search slope of the conjunction condition was significantly steeper than those of the orientation (*t*(14) = 9.98, *p* < .001) and colour search (*t*(14) = 8.60, *p* < .001) conditions. The search slope of the colour condition was also significantly steeper than that of the orientation condition (*t*(14) = 3.17, *p* < .01).

The search slopes of the target-absent trials were also analysed using repeated-measures ANOVA with breathing style × target-defining feature as factors. The analysis only showed a significant main effect of the target-defining feature (*F*(2, 28) = 86.62, *p* < .001, η_p_^2^ = .86). The search slope of the conjunction condition was significantly steeper than those of the orientation (*t*(14) = 10.42, *p* < .001) and colour (*t*(14) = 8.83, *p* < .001) conditions. However, the other main effects and interactions were not significant (*F*s < 1.61, *p*s > .18).

#### Intercepts

Two-way repeated-measures ANOVA of the intercepts of the target-present trials with breathing style × target-defining feature as factors revealed a significant main effect of the target-defining feature (*F*(2, 28) = 73.12, *p* < .001, η_p_^2^ = .84); however, the interaction (*F*(4, 56) = 0.22, *p* = .93, η_p_^2^ = .02) was not significant. Although the main effect of the breathing style (*F*(2, 28) = 2.66, *p* = .09, η_p_^2^ = .16) was not significant, the mean of the intercept of mouth breathing (*M*_log10_ = 2.75, *SD* = 0.10) was higher than those of the nasal breathing (*M*_log10_ = 2.74, *SD* = 0.10) and control (*M*_log10_ = 2.73, *SD* = 0.09) conditions. Multiple comparisons of the interaction indicated that the search intercept of the conjunction condition was significantly greater than those of the orientation (*t*(14) = 6.85, *p* < .001) and colour search (*t*(14) = 9.88, *p* < .001) conditions. The search intercept of the orientation condition was also greater than that of the colour condition (*t*(14) = 7.42, *p* < .001).

The search intercepts of the target-absent trials were analysed using repeated-measures ANOVA with breathing style × target-defining feature as factors. This analysis only showed a significant main effect of the target-defining feature (*F*(2, 28) = 15.59, *p* < .001, η_p_^2^ = .53). The search slope of the conjunction condition was significantly steeper than those of the orientation (*t*(14) = 2.48, *p* < .05) and colour (*t*(14) = 2.36, *p* < .05) conditions. The other main effects and interactions were not significant (*F*s < 1.60, *p*s > .22).

### Discussion

The aim of Experiment 1 was to investigate whether oral respiration negatively affects visual search performance. Although the search slope was adequately manipulated, breathing style did not influence the slope as an index of search efficiency. In addition, breathing style did not significantly influence the intercept; however, oral respiration heightened the intercept, which is an index of pre-search sensory processing and post-search response execution time. Although the data demonstrated a trend for the disruptive effects of oral respiration on the intercept, this was not statistically supported.

Breathing style effects may not have been visible because of methodological issues. First, there were only 15 participants in Experiment 1. In addition, the number of task trials per cell was less than those of typical visual tasks (e.g., Treisman & Gelade, 1980). Therefore, the small number of participants and trials might have resulted in an underpowered analysis. Our experiment may have required more participants and trials to make breathing style effects observable. In addition, target eccentricity was not strictly controlled in this experiment. The next experiment solved these problems and manipulated the search efficiency without changing the nature of the task, as in Liesefeld, Müller, Moran, Usher, and Zehetleitner (2016).

## Experiment 2

### Methods

#### Participants

Thirty-six graduate and undergraduate students at Kyushu University (30 women, mean age: 21.58 years, *SD* = 2.38, age range: 18–28) participated in the experiment. All participants gave written informed consent in accordance with the Declaration of Helsinki. They received a 1500-yen cash voucher as compensation. The ethics committee of Kyushu University approved the study protocol (approval number: 2017-004).

#### Apparatus and Stimuli

The surgical tape and nasal plug used in Experiment 2 were identical to those used in Experiment 1. Stimulus presentation and data collection were run on a Mac Mini (Apple, Cupertino, California) using a Matlab program (Mathworks, Natick, Massachusetts). Search displays were presented on a CRT monitor (1024 × 764, 100 Hz), and the participants maintained a viewing distance of 55 cm using a chin rest.

**Figure 5:**
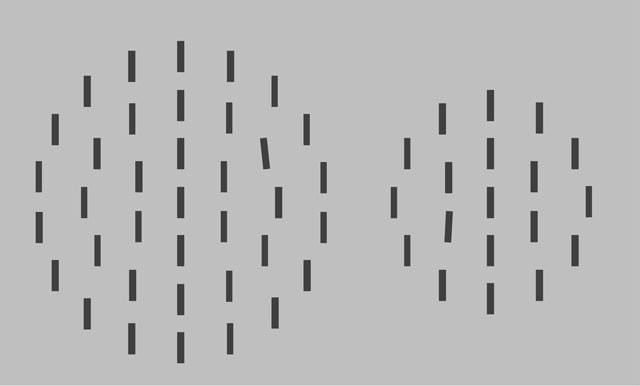
Sample displays for each set size from Experiment 2. The left array of stimuli indicates the display in which the target is tilted at 6° in set size 37. The right array of stimuli indicates the display in which the target is tilted at 4° in set size 19.

The stimuli were very similar to those of Liesefeld et al. (2016). The search items were dark grey bars (size, 1.35° × 0.037°) on a grey background (see Figure 5). The stimuli were either 19 or 37 bars (set size condition). Bars were arranged on two or three imaginary circles around a central bar with radii of 2.1°, 4.2°, and 6.3°. Half of the trials did not contain a tilted target bar among homogenous distractors (target-absent trials). In the remaining trials, a target bar that was tilted to the left or right was presented among homogenous distractors (target-present trials). The target bar was oriented to 4°, 5°, or 6° for each condition (target-distractor contrast). A previous study suggested that stimuli density affects target salience (Nothdurft, 2000). For this reason, the concentric stimulus arrangement was always maintained at a constant density. The stimuli on the outer circle were only used to make the target-distractor contrast comparable for all possible target positions. Another problem is that the display area of the array is different between large and small set sizes. Because target eccentricity affects target discriminability (Carrasco, Evert, Chang, & Katz, 1995), we manipulated the set size independently from the eccentricity. Specifically, we maintained the potential eccentricity of the target within the set size by randomly positioning the small-array search area within the large-array search area.

#### Procedure

The breathing condition examination procedure was identical to that of Experiment 1. The procedure was also very similar to Liesefeld et al. (2016). This experiment was conducted in a dark room. Participants were instructed to press the left arrow key if the target was present and right arrow key if the target was not present, or vice versa, which was counterbalanced across participants. When the participants pressed either the left or right arrow key, their RT was measured. If the response was incorrect, then a cross mark was immediately presented as feedback. Before a stimulus array was presented on the screen, a fixation cross was shown at random intervals between 700 ms and 1100 ms. Orientation contrasts (4°, 5°, and 6°) were blocked, with the order of blocks randomized for each participant. One block consisted of 144 experimental trials. Breaks were taken after every 30 trials. Participants conducted 16 practice trials for each block to become acquainted with the established orientation contrast. In the practice trials under the target-present condition, the target was always presented in the centre of the concentric array. In the experimental trials under the target-present condition, the target was presented within the array, except for the centre and outer ring positions. Participants performed three blocks of experimental trials (144 trials) per breathing style (repeated-measures design). Thus, 1296 trials were conducted.

#### Data Analysis

The dependent variables were log-transformed RTs. In each target-presence condition, only correct response data were used. Outliers were values that were 1.5 interquartile differences above the third quartile or below the first quartile of the respective empirical distribution, as in Experiment 1. RT_log10_ outliers were determined for each participant × breathing style × orientation contrast × set size cell and were excluded from correct RT_log10_ trials. Trial data in each target-presence condition were analysed using repeated-measures ANOVA with breathing style × orientation contrast × set size as factors.

The slopes and intercepts were also calculated as parameters measuring different aspects of visual search behaviour and were analysed using ANOVA. To extract the slopes and intercepts of each participant × orientation contrast × breathing style cell, we regressed the mean log-transformed RTs by the set size as RT = b1 + b2 × set size. Specifically, we calculated the lines connecting the mean RTs for the two set sizes. When calculating the slope, Liesefeld et al. (2016) did not include the circumference stimulus in the number of set sizes because participants may not have searched for them during the experiment. Therefore, the set size values included in the slope and intercept calculations were seven and 19 items. Mean slopes (ms/item) and intercepts (ms) from Experiment 2 are provided in Table 2.

**Table 2:** Mean Search Slopes (ms/item) and Intercepts (ms) with Standard Errors (in Brackets) in Experiment 2.

Slope						

	Present			Absent		

Breathing style	4°	5°	6°	4°	5°	6°

Control	0.54 (0.54)	–0.50 (0.26)	0.20 (0.24)	–0.25 (0.21)	0.22 (0.15)	–0.01 (0.09)
Nasal	0.14 (0.17)	–0.06 (0.17)	–0.12 (0.15)	0.12 (0.17)	–0.02 (0.09)	–0.02 (0.15)
Mouth	0.25 (0.16)	0.01 (0.10)	0.03 (0.11)	0.32(0.19)	0.21 (0.11)	0.14 (0.08)
**Intercept**						

Control	771.67 (45.17)	688.13 (35.57)	695.65 (36.68)	779.52 (45.18)	681.93 (33.40)	643.28 (26.47)
Nasal	747.04 (37.69)	730.79 (37.71)	645.7 (28.80)	748.99 (37.22)	732.79 (37.45)	644.47 (29.15)
Mouth	806.08 (45.81)	695.65 (36.68)	642.72 (30.48)	805.47 (46.87)	693.99 (36.09)	641.74 (30.36)

### Results

**Figure 6:**
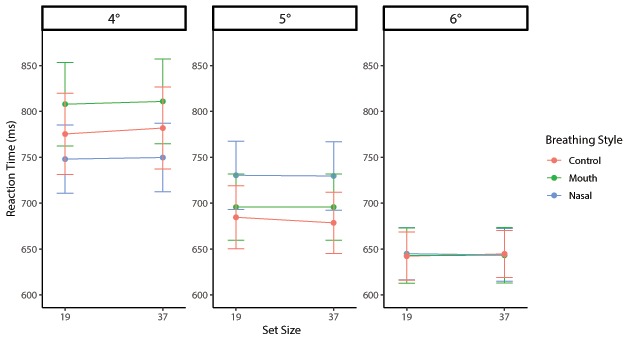
Mean RTs for each orientation contrast plotted as a function of breathing style and set size in target-present trials for Experiment 2. Error bars represent the standard error of the mean.

**Figure 7:**
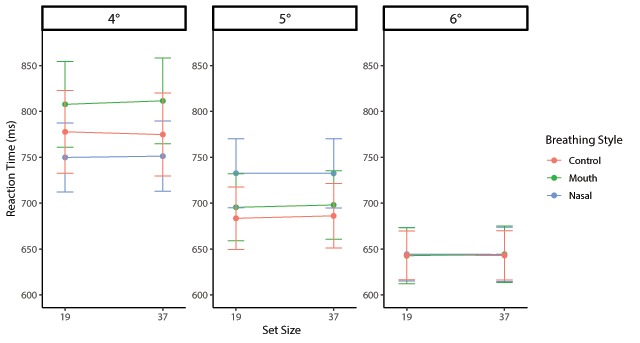
Mean RTs for each orientation contrast plotted as a function of breathing style and set size in target-absent trials for Experiment 2. Error bars represent the standard error of the mean.

The actual RTs are plotted in Figures 6 and 7 as an overview of the pattern of RTs. Repeated-measures ANOVA of the mean RT_log10_ during target-present trials found a significant main effect of the orientation contrast (*F*(2, 70) = 154.06 *p* < .001, η_p_^2^ = .81), with smaller target slopes resulting in slower searches (4°: *M*_log10_ = –0.17, *SD* = 0.09; 5°: *M*_log10_ = –0.23, *SD* = 0.09; 6°: *M*_log10_ = –0.26, *SD* = 0.07). The main effect of the set size was also significant (*F*(1, 35) = 75.80, *p* < .001, η_p_^2^ = .68), indicating that the RT of set size 37 was significantly slower than that of set size 19. However, there was no main effect of the breathing style (*F*(2, 70) = 0.87, *p* = .42, η_p_^2^ = .02). The interaction between the breathing style and orientation contrast was significant (*F*(4, 140) = 8.88, *p* < .001, η_p_^2^ = .20). Pairwise comparisons revealed that RT_log10_s in the 4° condition were slower in the oral respiration condition (*M*_log10_ = –0.16, *SD* = 0.10) than the control (*M*_log10_ = –0.19, *SD* = 0.09; *t*(35) = 3.02, *p* < .01) and nasal (*M*_log10_ = –0.18, *SD* = 0.08; *t*(35) = 2.52, *p* < .05) conditions. Although the simple main effect of the 5° condition was significant (*F*(2, 70) = 3.23, *p* < .05, η_p_^2^ = .08), no difference was observed between breathing style conditions (*t*s < 2.26, *p*s > .03). The simple main effect of the 6° condition was not significant (*F*(2, 70) = 0.55, *p* = 0.58, η_p_^2^ = .02). In addition, there was no significant difference between the control and nasal conditions at 4° (*t*(35) = 0.68, *p* = .49). Although the interaction between the breathing style and set size was significant (*F*(2, 70) = 4.08, *p* = .02, η_p_^2^ = .10), there was no difference in the breathing styles between the set size conditions. In addition, a significant interaction between the orientation contrast and set size (*F*(2, 70) = 3.19, *p* = .04, η_p_^2^ = .08) was observed; however, there was no triple interaction (*F*(4, 140) = 1.51, *p* = .20, η_p_^2^ = .04).

Repeated-measures ANOVA of mean RT_log10_ during target-absent trials revealed a significant main effect of the orientation contrast (*F*(2, 70) = 87.36, *p* < .001, η_p_^2^ = .71), with smaller target orientations associated with slower searches (4°: *M*_log10_ = –0.13, *SD* = 0.12; 5°: *M*_log10_ = –0.17, *SD* = 0.11; 6°: *M*_log10_ = –0.20, *SD* = 0.10). However, there were no main effects of the breathing style (*F*(2, 70) = 0.22, *p* = .79, η_p_^2^ = .01) and set size (*F*(1, 35) = 1.80, *p* = .18, η_p_^2^ = .05). An interaction between the breathing style and orientation contrast was significant (*F*(4, 140) = 4.11, *p* < .01, η_p_^2^ = .11). A simple main effect of the breathing style in each orientation contrast was not significant (*Fs* < 2.61, *p*s > .08). Since the overall error rate was very low (5.86%), a speed-accuracy trade-off was not observed.

#### Slopes

The search slopes of the target-present trials were analysed with repeated-measures ANOVA with breathing style × orientation contrast as factors. The analysis demonstrated significant main effects of the breathing style (*F*(2, 70) = 4.08, *p* < .05, η_p_^2^ = .10) and orientation contrast (*F*(2, 70) = 3.19, *p* < .05, η_p_^2^ = .08); however, the interaction was not significant (*F*(4, 140) = 1.51, *p* = .20, η_p_^2^ = .04). Post hoc comparisons indicated that the nasal respiration search slope was steeper than that of the control (*t*(35) = 2.76, *p* < .05). However, there was no difference between the other breathing conditions (*t*s < 1.58, *p*s > .12). Multiple comparisons of the orientation contrast showed no significant differences between any of the conditions (*t*s < 2.51, *p*s > .05).

The search slopes of the target-absent trials were also analysed using repeated-measures ANOVA with breathing style × orientation contrast as factors. This analysis demonstrated significant main effects of the orientation contrast (*F*(2, 70) = 83.38, *p* < .001, η_p_^2^ = .71), and the interaction was significant (*F*(4, 140) = 4.29, *p* < .01, η_p_^2^ = .11). However, the main effect of the breathing style was not significant (*F*(2, 70) = 1.59, *p* = .21, η_p_^2^ = .04). A simple main effect of the breathing style was significant at 4° (*F*(2, 70) = 6.00, *p* < .01, η_p_^2^ = .14) and 5° (*F*(2, 70) = 3.79, *p* < .05, η_p_^2^ = .09). The slope of the oral respiration condition was significantly steeper than that of the control condition (*t*(35) = 3.17, *p* < .01). Moreover, the nasal respiration slope was steeper than that of the control (*t*(35) = 2.24, *p* < .05.) in the 4° condition. The slope of the oral respiration condition was significantly steeper than that of the control condition (*t*(35) = 2.92, *p* < .01); however, there was no difference between the other breathing conditions.

#### Intercepts

Two-way repeated-measures ANOVA of the intercepts with breathing style × orientation contrast as factors showed a main effect of the orientation contrast (*F*(2, 70) = 67.24, *p* < .001, η_p_^2^ = .66), but no main effect of the breathing style (*F*(2, 70) = 0.18, *p* = .83, η_p_^2^ = .01). Although the interaction between the breathing style and orientation contrast was significant (*F*(4, 140) = 2.64, *p* < .05, η_p_^2^ = .07), for each orientation contrast, none of the simple main effects of the breathing style were significant (*F*s < 2.90, *p*s > .06). The mean intercept of the oral respiration condition (*M*_log10_ = –0.20, *SD* = 0.08) was higher than those of the nasal respiration (*M*_log10_ = –0.22, *SD* = 0.06) and control (*M*_log10_ = –0.21, *SD* = 0.08) conditions at 4°. Multiple comparisons of the orientation contrast indicated that the intercept of the 4° condition was greater than those of the 5° (*t*(35) = 6.19, *p* < .001) and 6° (*t*(35) = 12.76, *p* < .001) conditions. The intercept of the 5° condition was also greater than that of the 6° condition (*t*(35) = 4.69, *p* < .001).

Two-way repeated measures ANOVA of the intercepts of target-absent trials with breathing style × orientation contrast as factors showed a main effect of the orientation contrast (*F*(2, 70) = 84.91, *p* < .001, η_p_^2^ = .71), but no main effect of the breathing style (*F*(2, 70) = 0.11, *p* = .89, η_p_^2^ < .01). The interaction was significant (*F*(4, 140) = 4.06, *p* < .01, η_p_^2^ = .10). The simple main effect of the breathing style in the 4° condition was significant (*F*(2, 70) = 4.77, *p* < .05, η_p_^2^ = .12). The mean intercept of the oral respiration condition (*M*_log10_ = –0.07, *SD* = 0.15) was significantly higher than that of the control condition (*M*_log10_ = –0.13, *SD* = 0.11; *t*(35) = 3.04, *p* < .05); however, there were no differences between the other conditions.

### Discussion

As an improved version of Experiment 1, Experiment 2 examined whether oral respiration disrupts visual search performance. This experiment found longer RTs under the oral respiration condition than the nasal respiration and control conditions only at the lowest target discriminability. These results were due to the high intercept under the oral respiration condition, which suggests slowed sensory processing, decision making, or motor response due to inefficient and difficult visual search caused by mouth breathing. These results support our hypothesis that oral respiration and top-down attentional control could interact as a result of the activation of overlapping brain regions.

Under the nasal respiration condition, the search slope was steeper than that of the control condition, which suggests that the allocation of attention to each item was somehow slowed by nose breathing. However, given that there was no significant difference in the search slope between the oral and nasal respiration conditions, the effect of nose breathing seems to be negligible. Because the participants never made mouse breathing during the task, this unnatural situation might have artificially disrupted attentional allocation. Therefore, future investigations are needed to address this issue.

## General Discussion

In this study, we hypothesized that mouth breathing and top-down attentional control may interact because of the overlap of brain regions activated during these activities. We predicted that oral respiration disrupts visual search performance when top-down attentional control, or inefficient visual search, is required. In Experiment 1, the intercept under the oral respiration condition tended to be higher than those under the nasal respiration or control conditions, although this trend was not statistically significant.

Experiment 2 more strictly manipulated the search efficiency. Only when the target discriminability was lowest, the RTs under the oral respiration condition were longer than those under the nasal respiration and control conditions. These results are likely due to the high intercept under the oral respiration condition, which suggests the deterioration of pre-search sensory processing or post-search response execution time in difficult search situations. These results are consistent with our prediction that oral respiration negatively affects visual search performance during inefficient visual search. Converging evidence has shown that, compared with the nasal respiration and control conditions, the oral respiration condition decreases visual search performance (i.e., pre-search sensory processing or post-search response execution) when the search task requires many cognitive resources.

The present study demonstrated that oral respiration disrupted sensory processing or motor response during inefficient search for a poorly discriminable target. We speculate that cognitive resource consumption is possibly disruptive in this situation. Oral respiration can consume many cognitive resources, which is reflected by increased prefrontal brain activation (Sano et al., 2013). When participants orally respired during visual search, the visual system did not have sufficient resources to achieve a difficult cognitive task, which resulted in disrupted inefficient visual search performance. Indeed, such a disruptive effect was apparent when target discrimination was most difficult (i.e., under the 4° condition).

In Experiment 2, search efficiency unexpectedly decreased when the participants nasally respired. Given that this effect was not dependent on task difficulty and there was no significant difference in the search efficiency between the oral and nasal breathing conditions, the disruptive effect of nasal respiration seems to be negligible or absent. This finding may have occurred because the nasal respiration condition required participants to respire unnaturally by limiting oral respiration or causing discomfort because of the surgical tape placed over the mouth. Our results suggest that the disruptive effects of oral respiration are greater than those of nasal respiration.

Several study limitations must be considered. First, we did not assess whether the participants normally breathed through their noses or mouths and if they were able to adequately control their breathing. Through otorhinolaryngological evaluations of participants recruited from elementary schools and hospitals, Kuroishi et al. (2015) identified participants who exhibited chronic oral or nasal respiration. In this study, we were unable to otorhinolaryngologically assess whether participants chronically exhibited oral respiration. Habitual mouth breathers may react differently to nose plugs than nasal breathers. Thus, information from participants concerning long-term breathing habits would provide a deeper understanding of the effect of breathing style on visual search tasks. Moreover, the present findings could possibly be related to physiological differences in the amount of air respired. Thus, future studies must consider chronic mouth breathing and monitor real-time breathing via air plethysmography.

## Data Accessibility Statement

Anonymized datasets are publicly available at the Open Science Framework (https://osf.io/cjz79/).

## References

[1] Anderson, E. J., Mannan, S. K., Husain, M., Rees, G., Sumner, P., Mort, D. J., McRobbie, D., & Kennard, C. (2007). Involvement of prefrontal cortex in visual search. Experimental Brain Research, 180(2), 289–302. DOI: 10.1007/s00221-007-0860-0 17310377

[2] Arch, J. J., & Craske, M. G. (2006). Mechanisms of mindfulness: Emotion regulation following a focused breathing induction. Behaviour Research and Therapy, 44(12), 1849–1858. DOI: 10.1016/j.brat.2005.12.007 16460668

[3] Awh, E., Belopolsky, A. V., & Theeuwes, J. (2012). Top-down versus bottom-up attentional control: A failed theoretical dichotomy. Trends in Cognitive Sciences, 16(8), 437–443. DOI: 10.1016/j.tics.2012.06.010 22795563 PMC3426354

[4] Bacon, W. F., & Egeth, H. E. (1994). Overriding stimulus-driven attentional capture. Perception & Psychophysics, 55(5), 485–496. DOI: 10.3758/BF03205306 8008550

[5] Bichot, N. P., Heard, M. T., DeGennaro, E. M., & Desimone, R. (2015). A source for feature-based attention in the prefrontal cortex. Neuron, 88(4), 832–844. DOI: 10.1016/j.neuron.2015.10.001 26526392 PMC4655197

[6] Buschman, T, J., & Miller, E, K. (2007). Top-down versus bottom-up control of attention in the prefrontal and posterior parietal cortices. Science, 315(5820), 1860–1862. DOI: 10.1126/science.1138071 17395832

[7] Carrasco, M., Evert, D. L., Chang, I., & Katz, S. M. (1995). The eccentricity effect: Target eccentricity affects performance on conjunction searches. Perception & Psychophysics, 57(8), 1241–1261. DOI: 10.3758/BF03208380 8539099

[8] de Fockert, J. D., Rees, G., Frith, C., & Lavie, N. (2004). Neural correlates of attentional capture in visual search. Journal of Cognitive Neuroscience, 16(5), 751–759. DOI: 10.1162/089892904970762 15200703

[9] de Fockert, J. W., & Theeuwes, J. (2012). Role of frontal cortex in attentional capture by singleton distractors. Brain and Cognition, 80(3), 367–373. DOI: 10.1016/j.bandc.2012.07.006 22959916

[10] de Leeuw, J. R. (2015). jsPsych: A JavaScript library for creating behavioral experiments in a web browser. Behavior Research Methods, 47(1), 1–12. DOI: 10.3758/s13428-014-0458-y 24683129

[11] Jefferson, Y. (2010). Mouth breathing: Adverse effects on facial growth, health, academics, and behavior. General Dentistry, 58(1), 18–25. 20129889

[12] Kuroishi, R. C. S., Garcia, R. B., Valera, F. C. P., Anselmo-Lima, W. T., & Fukuda, M. T. H. (2015). Deficits in working memory, reading comprehension and arithmetic skills in children with mouth breathing syndrome: Analytical cross-sectional study. Sao Paulo Medical Journal, 133, 78–83. DOI: 10.1590/1516-3180.2013.7630011 25271880 PMC10496631

[13] Liesefeld, H. R., Moran, R., Usher, M., Müller, H. J., & Zehetleitner, M. (2016). Search efficiency as a function of target saliency: The transition from inefficient to efficient search and beyond. Journal of Experimental Psychology: Human Perception and Performance, 42(6), 821 DOI: 10.1037/xhp0000156 26727018

[14] Maljkovic, V., & Nakayama, K. (1994). Priming of pop-out: I. Role of features. Memory & Cognition, 22(6), 657–672. DOI: 10.3758/BF03209251 7808275

[15] Martin, M. S., Sforza, E., Roche, F., Barthelemy, J. C., & Thomas-Anterion, C. (2015). Sleep breathing disorders and cognitive function in the elderly: An 8-year follow-up study. the proof-synapse cohort. Sleep, 38, 179–187. DOI: 10.5665/sleep.4392 25325480 PMC4288598

[16] Miller, E. K., & Buschman, T. J. (2013). Cortical circuits for the control of attention. Current Opinion in Neurobiology, 23(2), 216–222. DOI: 10.1016/j.conb.2012.11.011 23265963 PMC3709832

[17] Nakamura, N. H., Fukunaga, M., & Oku, Y. (2018). Respiratory modulation of cognitive performance during the retrieval process. PLOS ONE, 13(9): e0204021 DOI: 10.1371/journal.pone.0204021 30216372 PMC6138381

[18] Nothdurft, H. C. (2000). Salience from feature contrast: Variations with texture density. Vision Research, 40(23), 3181–3200. DOI: 10.1016/S0042-6989(00)00168-1 11008137

[19] Pevernagie, D. A., De Meyer, M. M., & Claeys, S. (2005). Sleep, breathing and the nose. Sleep Medicine Reviews, 9(6), 437–451. DOI: 10.1016/j.smrv.2005.02.002 16242364

[20] Sano, M., Sano, S., Oka, N., Yoshino, K., & Kato, T. (2013). Increased oxygen load in the prefrontal cortex from mouth breathing: A vector-based near-infrared spectroscopy study. NeuroReport, 24(17), 935–940. DOI: 10.1097/WNR.0000000000000008 24169579 PMC4047298

[21] Shimamura, A. P. (2000). The role of the prefrontal cortex in dynamic filtering. Psychobiology, 28(2), 207–218.

[22] Theeuwes, J. (1992). Perceptual selectivity for color and form. Perception & Psychophysics, 51(6), 599–606. DOI: 10.3758/BF03211656 1620571

[23] Theeuwes, J. (2010). Top–down and bottom–up control of visual selection. Acta Psychologica, 135(2), 77–99. DOI: 10.1016/j.actpsy.2010.02.006 20507828

[24] Theeuwes, J. (2018). Visual selection: Usually fast and automatic; Seldom slow and volitional. Journal of Cognition, 1(1), 21 DOI: 10.5334/joc.32 PMC6634613 31517202

[25] Treisman, A., & Gelade, G. (1980). A feature-integration theory of attention, Cognitive Psychology, 12(1), 97–136. DOI: 10.1016/0010-0285(80)90005-5 7351125

[26] Vecera, S. P., Cosman, J. D., Vatterott, D. B., & Roper, Z. J. (2014). The control of visual attention: Toward a unified account In Psychology of Learning and Motivation, 60, 303–347. Academic Press DOI: 10.1016/B978-0-12-800090-8.00008-1

[27] Wolfe, J. M., & Horowitz, T. S. (2017). Five factors that guide attention in visual search. Nature Human Behaviour, 1(3), 0058 DOI: 10.1038/s41562-017-0058 PMC9879335 36711068

[28] Young, T., Finn, L., & Kim, H. (1997). Nasal obstruction as a risk factor for sleep-disordered breathing. Journal of Allergy Clinical Immunology, 99(2), 757–762. DOI: 10.1016/S0091-6749(97)70124-6 9042068

[29] Zautra, A. J., Fasman, R., Davis, M. C., & Arthur, D. (2010). The effects of slow breathing on affective responses to pain stimuli: An experimental study. Pain, 149(1), 12–18. DOI: 10.1016/j.pain.2009.10.001 20079569

[30] Zelano, C., Jiang, H., Zhou, G., Arora, N., Schuele, S., Rosenow, J., & Gottfried, F. A. (2016). Nasal respiration entrains human limbic oscillations and modulates cognitive function. The Journal of Neuroscience, 36(49), 12448–12467. DOI: 10.1523/JNEUROSCI.2586-16.2016 27927961 PMC5148230

